# Transumbilical Surgery for Duodenal Stenosis in a Child with Situs Inversus: The First Report

**DOI:** 10.1155/2017/2074387

**Published:** 2017-03-12

**Authors:** Isamu Saeki, Yu Ueno, Wataru Mukai, Reisuke Imaji, Takashi Akiyama

**Affiliations:** ^1^Department of Pediatric Surgery, Hiroshima City Hospital, 7-33 Motomachi Naka-ku, Hiroshima-shi, Hiroshima 730-8518, Japan; ^2^Chugoku-Shikoku Pediatric Surgical Partners Organization, Japan

## Abstract

*Background*. Situs inversus is a rare congenital anomaly with a reported incidence of only 1 in 5,000 to 10,000 live births. Congenital duodenal stenosis complicated with situs inversus is an even rarer entity.* Case Presentation*. A 1-year-old girl with situs inversus who had undergone a hemi-Fontan procedure against a single ventricle in our hospital was referred to our department for vomiting and failure to thrive. An upper gastrointestinal contrast study and endoscopy revealed duodenal stenosis. A transumbilical radical operation as a minimally invasive surgery was successfully performed. After the surgery, she stopped vomiting, and the postoperative course was uneventful with good cosmetic results.* Conclusions*. To our knowledge, this is the first report of transumbilical surgery for congenital duodenal stenosis with situs inversus as minimally invasive surgery. Transumbilical surgery to situs inversus patient can be performed safely and lead to good cosmetic outcome.

## 1. Introduction

Situs inversus (SI) is a rare clinical malformation with an incidence of 1 in 5,000 to 10,000 live births [1]. The surgical procedures for SI patients are technically challenging because of the mirror image anatomical construction and high frequency of other anomalies. Congenital duodenal obstruction, which includes atresia and stenosis, is estimated to have an incidence of about 1 in 4,000 to 15,000 live births [2, 3]. Congenital duodenal stenosis (CDS) is relatively rare in comparison with duodenal atresia and sometimes has a delayed presentation [3–5]. CDS associated with SI is an extremely rare entity and only a few cases have been reported thus far [2, 6]. We present a case of a 1-year-old girl with CDS and SI who successfully underwent a transumbilical radical operation as minimally invasive surgery.

## 2. Case Presentation

**Figure 1:**
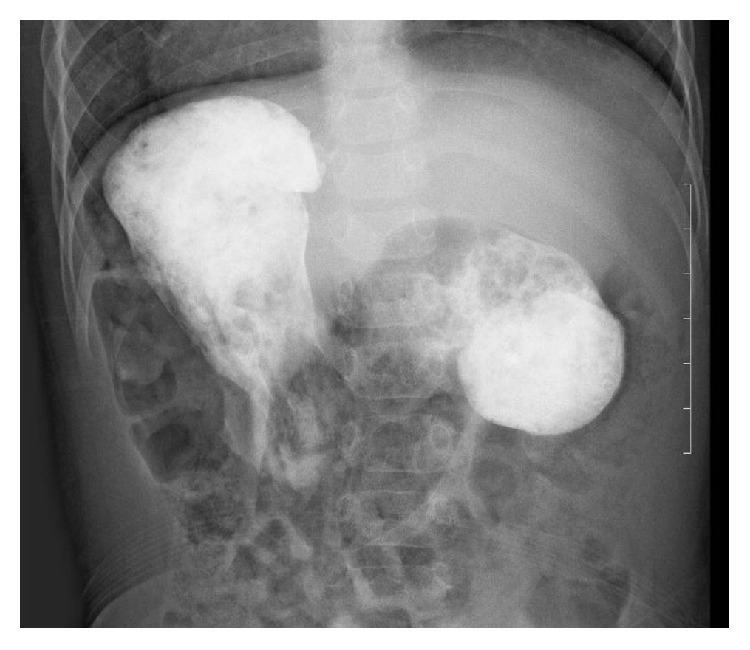
The upper gastrointestinal contrast study finding (supine position). The proximal duodenum was markedly dilated, which suggested duodenal obstruction.

**Figure 2:**
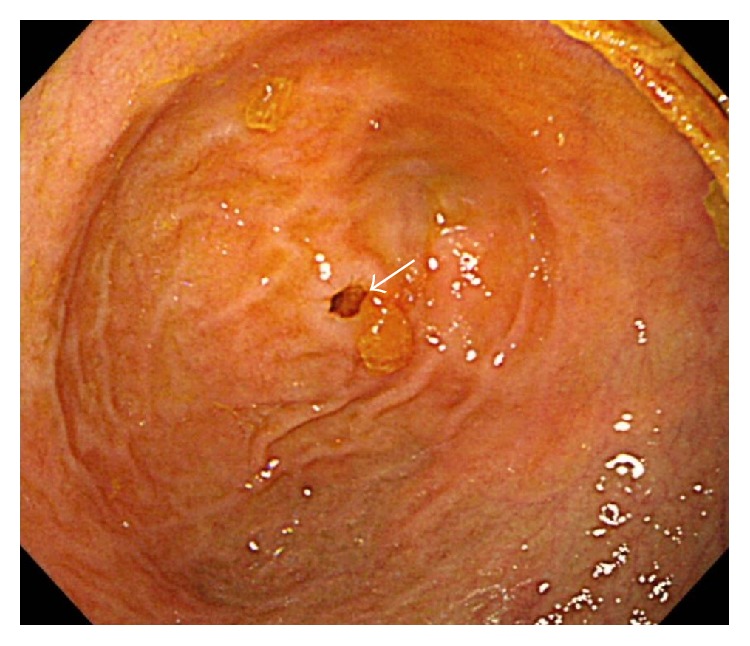
The gastroduodenal endoscopy findings. A pin-hole obstruction about 2 mm in diameter was found at the second portion of the duodenum (arrow).

A 1-year-old Asian girl who had undergone a hemi-Fontan procedure against a single ventricle in our hospital was referred to our department. She suffered from vomiting after feeding several times a day, and her body weight gain was slow. Her vomiting worsened after starting baby food. After the introduction of medication (mosapride citrate and rikkunshito, a Japanese traditional Kampo medicine) to improve the gastric function, her body weight gain improved slightly, but her vomiting persisted. An upper gastrointestinal contrast study showed marked dilation of the proximal duodenum which suggested duodenal obstruction (Figure 1). A gastroduodenal endoscopy revealed a pin-hole obstruction of about 2 mm in diameter at the second portion of the duodenum (Figure 2). Endoscopic dilation of the stenosis was abandoned because the location of the papilla Vater could not be identified. She instead underwent surgery at 22 months of age. Although transumbilical operations are usually restricted to neonates or infants, we selected this procedure because the patient's physical size was not very big for her age.

### 2.1. Operative Procedures

**Figure 3:**
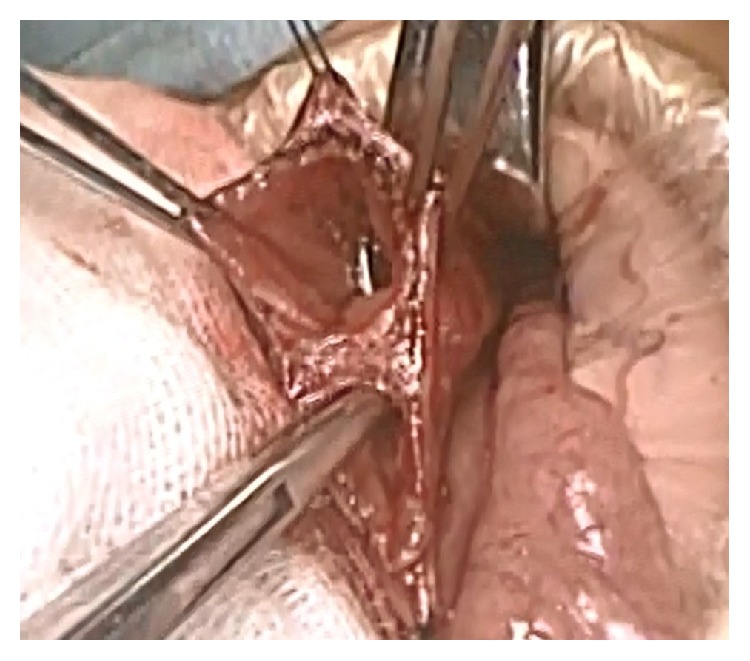
The surgical findings. The longitudinal incision of the duodenum revealed a thick web stenosis of the duodenum with a very small central hole.

An upper 2/3 circumference incision of the umbilicus was made, and a left transverse incision 1.5 cm in diameter was added continuously. The linea alba was dissected transversely for laparotomy, and hepatic round ligament was also dissected to increase the size of the operative field. An Alexis® wound retractor XS (Applied Medical, Santa Rancha Margarita, CA, USA) was inserted through the umbilical wound, and the Kocher maneuver was performed. A caliber change in the duodenum was detected at the second portion. The longitudinal incision of the duodenum revealed a thick web stenosis of the duodenum with a very small central hole (Figure 3). The papilla Vater was not located around the web. After the dissection of the web, a 6.5Fr New enteral feeding tube (Kangaroo™ Covidien, Japan) was inserted into the jejunum as a transanastomotic tube, and the duodenum was closed transversely. No other intestinal anomalies such as malrotation were detected. The operation time was 107 minutes, with little operative hemorrhaging.

**Figure 4:**
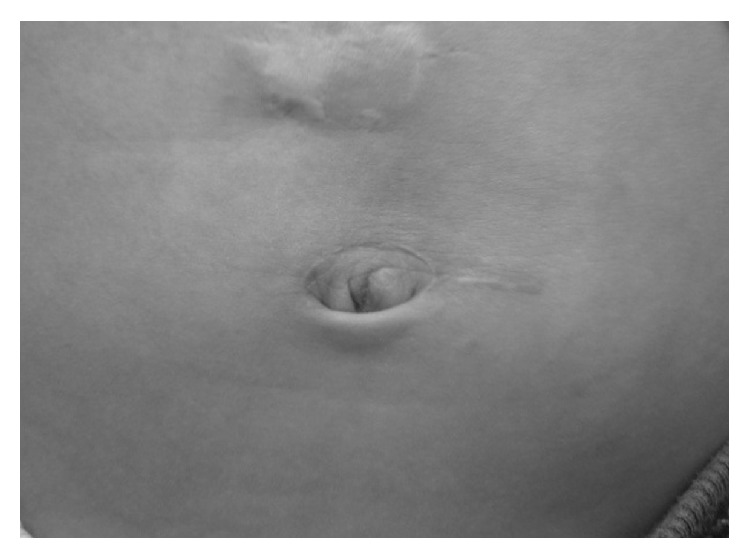
Wound scar at one year after surgery. The cosmetic outcome was good at the one-year follow-up.

The postoperative clinical course was uneventful. Enteral feeding through transanastomotic tube was started a day after surgery and she was allowed food seven days after surgery. She was discharged nine days after surgery without vomiting. The body weight gain after surgery was favorable, and the cosmetic outcome is good after 1 year of follow-up (Figure 4).

## 3. Discussion

CDS is a relatively rare condition in comparison with congenital duodenal atresia [4]. Due to the incomplete obstruction of the duodenum, CDS often has a delayed presentation, which makes a diagnosis difficult [2–4]. A fluid diet like milk can pass through the duodenal stenosis but not solid food. Vomiting is the most common symptom in infants with CDS, and similarly, in our case, the vomiting worsened after starting baby food.

Congenital duodenal obstruction associated with SI is an extremely rare condition, and only about 20 patients have been reported in the literature [2, 6, 7]. In particular, only a few CDS patients associated with SI have ever been reported before [2]. Although there are some reports of gastroscopic treatment for membranous duodenal stenosis, we selected open surgery because the location of the papilla Vater could not be identified in our case [5, 8].

Surgical procedures in SI patients are challenging due to the mirror image presentation and associated malformations; therefore, such procedures should be performed with a good surgical view by practiced surgeons [9]. An increasing number of reports have been published in recent years regarding laparoscopic surgery in SI patients [10–12]. In particular, a technical review was conducted regarding the outcomes of laparoscopic cholecystectomy [12]. Laparoscopic surgery has a great surgical advantage over open surgery due to its minimally invasiveness. However, the rarity and the unique features of SI, such as mirror image and its many associated malformations, hamper operations. In addition, an inadequate viewing field may increase the risk of the procedure. A careful preoperative assessment is necessary when performing minimally invasive surgery in SI patients.

Transumbilical surgery for neonatal congenital duodenal obstruction is reported to be a safe and useful therapeutic modality with a relatively large operating field and good cosmetic results. Tajiri et al. [13] precisely described how to obtain a good surgical view when using the umbilical approach (citation), as follows. To obtain a large operative field, opening the fascia upward in the midline, a slight transverse cut of the right rectus muscle and a cut of the hepatic round ligament in some cases with an incision on its upper half circumference are very important maneuvers. The procedure is minimally invasive as it does not require rectus abdominis muscle dissection compared to transverse incision of the upper abdomen. At our institution, almost all of the congenital duodenal obstruction patients are operated via the transumbilical approach unless they have a severe general condition. Although there have been no reports of transumbilical surgery in a CDS child associated with SI before, we were able to perform the operation safely involving a number of surgeons accustomed to transumbilical surgery. Because the patient was 1 year old and physically bigger than neonates, a left transverse incision 1.5 cm in diameter was added to the umbilical incision, but the cosmetic outcome remained excellent.

## 4. Conclusion

We performed transumbilical surgery for CDS in a child with SI as a minimally invasive surgery and obtained good cosmetic results.

## Abbreviations

CDS: Congenital duodenal stenosis
SI: Situs inversus.
